# Focal Adhesion’s Role in Cardiomyocytes Function: From Cardiomyogenesis to Mechanotransduction

**DOI:** 10.3390/cells13080664

**Published:** 2024-04-10

**Authors:** Simona Casarella, Federica Ferla, Dalila Di Francesco, Elena Canciani, Manuela Rizzi, Francesca Boccafoschi

**Affiliations:** 1 Department of Health Sciences, University of Piemonte Orientale, 28100 Novara, Italy; simona.casarella@uniupo.it (S.C.); dalila.di-francesco.1@ulaval.ca (D.D.F.); elena.canciani@uniupo.it (E.C.); manuela.rizzi@med.uniupo.it (M.R.); 2 Laboratory for Biomaterials and Bioengineering, CRC-I, Department of Min-Met-Materials Engineering, University Hospital Research Center, Regenerative Medicine, Laval University, Quebec City, QC G1V 0A6, Canada

**Keywords:** focal adhesions, integrins, ECM, mechanotransduction, cardiomyocytes, FAK

## Abstract

Mechanotransduction refers to the ability of cells to sense mechanical stimuli and convert them into biochemical signals. In this context, the key players are focal adhesions (FAs): multiprotein complexes that link intracellular actin bundles and the extracellular matrix (ECM). FAs are involved in cellular adhesion, growth, differentiation, gene expression, migration, communication, force transmission, and contractility. Focal adhesion signaling molecules, including Focal Adhesion Kinase (FAK), integrins, vinculin, and paxillin, also play pivotal roles in cardiomyogenesis, impacting cell proliferation and heart tube looping. In fact, cardiomyocytes sense ECM stiffness through integrins, modulating signaling pathways like PI3K/AKT and Wnt/β-catenin. Moreover, FAK/Src complex activation mediates cardiac hypertrophic growth and survival signaling in response to mechanical loads. This review provides an overview of the molecular and mechanical mechanisms underlying the crosstalk between FAs and cardiac differentiation, as well as the role of FA-mediated mechanotransduction in guiding cardiac muscle responses to mechanical stimuli.

## 1. Focal Adhesions: Dynamic Sites Involved in Cell Adhesion and Function

Focal adhesions (FAs) are protein complexes that mediate cell adhesion by connecting the cytoskeleton to the extracellular matrix. They were first identified in the 1970s by Abercrombie and colleagues with electron microscopy, and since then, over 50 proteins associated with FAs have been reported [1]. FAs contribute to cell migration, translating forces on actin fibers known as stress fibers. These stress fibers are specialized forms of F-actin associated with myosin II filaments, crosslinked by alpha-actinin and other associated proteins, such as integrins’ transmembrane receptors: α- and β-integrins. There are different types of stress fibers associated with FAs: ventral stress fibers are mainly at the extremities of FAs and cross through the whole cell; dorsal stress fibers are typically present on one extremity of FAs, and they extend up to the nucleus and the dorsal cell surface [2,3,4,5,6].

During FA assembly, integrin transmembrane receptors bind to the ECM, an event that induces the clustering of integrins and conformational changes; then, other adaptor proteins, such as paxillin and talin, are recruited, inducing the activation of integrins and gathering actin stress fibers [5,6]. In general, FAs are highly dynamic and hierarchical complexes, starting from the bottom and middle layers to the top, interacting with actin-binding proteins [5,7,8]. Paxillin is a multidomain scaffold protein that interacts directly with integrins and facilitates the recruitment and interaction of other proteins involved in FA development. One of the first proteins that paxillin recruits directly is Focal Adhesion Kinase (FAK), a non-receptor tyrosine kinase which auto-phosphorylates in Tyr397 and, in turn, phosphorylates Src and paxillin in a mechanosensitive way, influencing FA size [9,10,11]. During the formation of FAs, zyxin and vasodilator-stimulated protein (VASP) proteins are recruited, and they mediate the coupling of actin filaments, allowing for the formation and extension of stress fibers containing the α-actinin binding site, specifically required for actin binding [5,8]. The middle layer of FAs is composed of talin and vinculin proteins. Talin is an integrin-associated protein that binds directly to β-integrins through a globular head domain, stimulating integrin activation; the talin tail domain, instead, directly binds to actin filaments and forms several vinculin-binding sites, also enhancing vinculin activation [5,12,13]. Studies suggest that this dynamic assembly and disassembly of the cytoskeleton plays a crucial role in cellular differentiation and migration during cardiac development and may also be an important regulatory factor during new sarcomere addition in response to hypertrophic stimuli [8].

Interestingly, cardiomyocytes have two different types of FAs: peripheral focal adhesions (pFAs), laterally associated with myofibrils, and costameres, associated with sarcomeres [14,15]. The term ‘costamere’ was first used by Craig and colleagues to describe vinculin-containing, rib-like bands that encircle cardiomyocytes perpendicularly to their long axis [16,17]. Costameres, as the non-muscle cells’ FAs, consist of a complex protein network forming a physical link between the ECM and the outer Z-discs of cardiomyocytes through the integrins and the dystrophin–glycoprotein complex, which mediates the attachment to the ECM protein laminin [18,19]. Moreover, costameres transmit both external and internal mechanical loads; they directly transmit contractile forces generated within the cardiomyocyte to the surrounding ECM and from adjacent muscle cells’ ECM to the internal contractile mechanism [18,20]. However, it has been demonstrated how defects or mutations in FA proteins may lead to cardiomyopathies, thus revealing the importance of costameres in normal cardiac function and myocardial remodeling [21].

## 2. Focal Adhesions and Cardiac Cell Differentiation

Studies in the literature report that cardiomyocytes initiate myofibrillar assembly at the outer region of the cell, starting from pre-myofibrils composed of non-muscle myosin II (NMM II) and α-actinin-2 fibers. This initial assembly guides the subsequent incorporation of titin, α-cardiac myosins, and/or β-cardiac myosins, leading to the formation of fully developed myofibrils [22,23]. The evidence that cardiac myofibrillar assembly originates at the cell periphery has also supported the hypothesis implicating protocostameres, which resemble FAs and serve as sites of cell–ECM adhesion [24]. Protocostameres share a molecular composition similar to classical FAs and encompass proteins such as integrins, paxillin, vinculin, and FAK. Over time, protocostameres mature into specialized cell–ECM junctions known as costameres, which coincide with the Z-disks at the plasma membrane [11]. Furthermore, increasing attention is being paid to the crucial role of myosin-generated force in myofibrillar assembly in both skeletal and cardiac muscles, both in vivo and in vitro [25,26,27].

During heart development, sarcomeres undergo dynamic remodeling processes crucial for the maturation and functional adaptation of the myocardium. These processes involve modifications in terms of the composition of sarcomeric proteins like myosin, actin, and titin, which directly influence the contractile properties of cardiomyocytes [28,29]. A study conducted by Kresh and Chopra demonstrated that changes in sarcomere structure and organization directly affect cardiomyocyte force generation. The altered sarcomere architecture in disease conditions thus leads to impaired contractile function, contributing to the compromised cardiac performance observed in heart diseases [30].

Moreover, the presence of full-length titin is necessary for generating basal tension in cardiomyocytes [31]. Titin spans from the Z-disc to the M-line within the sarcomere and acts as a molecular spring, providing elasticity and contributing to the passive stiffness of cardiomyocytes [31,32]. Chopra and colleagues argue that this basal tension is a prerequisite for the initiation of sarcomere assembly [31]. Furthermore, the study revealed that β-cardiac myosin, a specific isoform of myosin found in the cardiac muscle, plays a critical role in generating the required basal tension for directing sarcomere assembly. The researchers showed that the mechanical link between β-cardiac myosin and titin is essential for generating tension and subsequently initiating sarcomere assembly. The study also proves the significance of protocostameres in the assembly of sarcomeres [31]. The coupling of titin to protocostameres was found to be crucial for driving the sarcomere assembly process. This suggests that the mechanical connection between titin and protocostameres plays a pivotal role in transmitting the tension required for initiating sarcomere assembly at the cell periphery [31,33]. Their findings highlighted the mechanical and molecular mechanisms underlying the generation of basal tension and its role in directing sarcomere assembly. This knowledge contributes to understanding cardiomyocyte development and may have implications for studying cardiac disorders associated with sarcomere assembly defects [34].

Additionally, the interplay between FA signaling and cytoskeletal remodeling pathways is crucial for coordinating myofibrillar assembly and maturation during cardiomyogenesis. At the molecular level, during cardiomyocyte differentiation, the maturation process of FAs plays an important role in activating different pathways and genes, such as integrin/FAK/PI3K-P85, which is activated by the interaction between laminins and early growth response protein 1 (ERG1), forming a complex with b1D integrin. The consecutive phosphorylation of FAK and PI3K-P85 activates AKT, inhibiting Wnt-GSK3b, which, in turn, upregulates β-catenin and GATA-4, necessary for cardiac differentiation [35,36,37,38].

A study by Doherty and colleagues investigated the role of FAK in cardiac looping, a critical process during heart formation [39]. By suppressing FAK expression, the researchers observed a series of detrimental effects on cardiac development. First, they noted a reduction in mitotic activity, indicating a diminished ability of cardiomyocytes to undergo cell division. This finding suggests that FAK plays a crucial role in promoting cell proliferation during the early stages of cardiogenesis [40,41,42,43]. Furthermore, the study showed that FAK depletion resulted in a failure of heart tube looping. Cardiac looping is a critical morphogenetic event in which the linear heart tube undergoes a bending and twisting process to acquire its characteristic looping structure [44]. The failure of heart tube looping observed in FAK-depleted embryos indicates that FAK is essential for this key step in cardiac development [39].

As mentioned before, during the formation of FAs, the phosphorylation of FAK-Tyr397 has a crucial role in myocardial development: FAK auto-phosphorylation induces a signaling cascade, with a consecutive survival promotion through Erk1/2, S6K, mTORC1, and Akt activation [45,46,47]. Moreover, FAK can be phosphorylated by the interaction with the heterodimer ErbB2/ErbB4 and Nrg1β, recruiting Src, which, in turn, phosphorylates residues Tyr861 and Tyr925, modulating cell survival, invasion, and cell–cell interaction [45].

Another relevant protein for FA maturation and cytoskeleton protein interactions in cardiomyocytes is CASK, which, with its HOOK domain, forms the complex Mint1/Veli/SAP97/CASK, which interacts with dystrophin–glycoprotein and gives structural and functional support to the sarcomeres [48]. Moreover, VEGF enhances the adhesion of contractile cells to the ECM through the activation of p125FAK, with consequent paxillin phosphorylation, which then interacts with LIM–nebulette, enhancing cardiomyocyte adhesion [49]. The N-terminal domain of the adaptor protein paxillin possesses a proline-rich region (PRR), which binds to the second SH3 domain of ponsin, another adaptor protein belonging to the vinexin protein family. This interaction happens after the onset of myogenic differentiation and the onset of the maturation of costameres, suggesting an important function of paxillin in cytoskeletal remodeling and costamerogenesis [11,50,51].

Talin, vinculin, and tensin1 are FA structural proteins that provide a physical link between the integrins and the actin cytoskeleton [52,53,54]. When phosphorylated and thus activated, talin recruits vinculin, and together, these proteins bind to the integrin–actomyosin system, leading to the maturation and stabilization of FAs and costameres [55,56,57,58]. Moreover, vinculin supports three other different recruitment and activation mechanisms [52]. The first mechanism suggests that vinculin interacts with two distinct components: through its head domain, vinculin binds talin, and via the tail domain, it binds phosphatidylinositol 4,5-bisphosphate (PIP2), which activates the dimerization, increasing the actin binding to the tail and, subsequently, activating other components participating in the adhesion complex [59,60]. Otherwise, vinculin activation is based on conformational changes. Another approach shows that vinculin undergoes a transition from an inactive to an active state, allowing it to bind cytoplasmic talin and to form a cytoplasmic pre-complex, which is then recruited to integrin-bound sites, continuing the focal contact differentiation process [53,61]. In this last case, paxillin, when phosphorylated by FAK, transiently recruits vinculin, and the vinculin–paxillin complex interacts with talin, leading to the formation of a vinculin–talin bond [62,63].

During the formation of costameres, an additional fundamental element, myocyte enhancer factor 2A (Mef2A), is involved [64]. Mef2A belongs to a family of transcription factors that regulate muscle differentiation, and it is crucial for maintaining structural integrity and supporting cell survival during the early stages of costamere differentiation [65]. Following positive regulation through FAK-mediated phosphorylation, Mef2A exerts transcriptional control over different genes encoding proteins localized within the cytoskeletal structure. This regulation ensures the proper expression of individual protein components, ultimately enabling the correct structural development of adhesions and, consequently, the normal functioning of cardiac muscle [64,66].

The importance of the specific interaction between vinculin and talin becomes evident as it underlies the focal contact differentiation process. Table 1 provides an overview of the role of FAs in cardiac differentiation and maturation.

## 3. Focal Adhesion-Mediated Mechanosensing in Cardiac Muscle

Resident cells in tissues are constantly subjected to several mechanical stimuli that affect the homeostasis of the ECM and cell behavior through specialized cell–ECM interactions (e.g., FAs). The ability of cells to sense (mechanosensing) and respond (mechanosignaling) to these external stimuli, transducing them into biochemical, intracellular signals, is named mechanotransduction, which involves mechanosensing and mechanosignaling [20,67].

Cardiomyocytes are exposed to different types of forces essential for their development as well as for their physiological functions, including stretching and twisting forces deriving from contractions, hemodynamic pressure, and ECM-related passive elasticity. In this context, the composition of the ECM undergoes spatial and transient modifications during cardiomyogenesis, especially regarding the expression of laminins, collagens, matrix proteases, hyaluronan, and proteoglycans [68,69]. Several in vitro studies have shown how the ECM’s composition and elasticity (or stiffness) influences the cardiac contractile apparatus. As an example, Jacot and colleagues studied matrices with heart-like stiffness and demonstrated their ability to support the optimal contraction of neonatal rat ventricular myocytes (NRVMs). Moreover, changes in substrate stiffness affected contraction force, demonstrating the importance of ECM properties [70]. The composition and elasticity of the ECM also impact the organization of the contractile system within cardiomyocytes. Geisse and colleagues demonstrated that culturing neonatal rat cardiomyocytes on different micropatterned fibronectin islands led to distinct myofibril distribution patterns, highlighting the ECM’s influence on cytoskeletal architecture rearrangement [71]. Furthermore, studies on NRVMs have shown different cardiac maturation rates based on the type of ECM substrate [72,73]. A fibroblast-derived ECM was found to support the early cardiac differentiation of embryonic stem cells, as evidenced by spontaneous contractions, efficient calcium handling, changes in cell size, and mitochondrial development [74]. Notably, ECM component affinities differed across stages of heart development, indicating a role in developmental regulation [73,75].

Mechanotransduction processes are sensitive to changes in shear stress, cell adhesion forces, substrate rigidity, membrane or cytoskeletal stretching, and compression [76]. The transmission of cardiac mechanical stimuli involves a complex interplay between focal adhesions, intercalated discs, sarcomeres, costameres, the ECM, and the cytoskeleton. These cellular structures and mechanisms work together to sense and respond to mechanical forces, thereby regulating cardiac function and adaptation [16,77]. Different processes and mechanosensors are involved in cardiac mechanotransduction, but the pathways are not fully understood [78].

**Figure 1:**
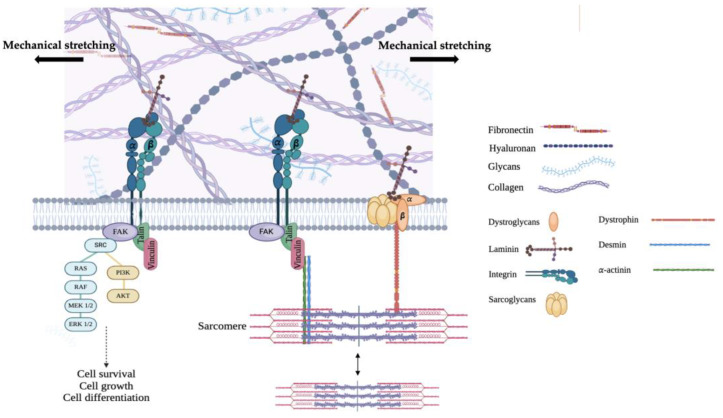
Schematic representation of inside-out and outside-in signaling in response to mechanical stress, mediated by integrins and other FA proteins such as vinculin and talin, and how they link the sarcomere to the ECM. Illustrated are some of the pathways activated by the phosphorylation of FAK-Src that involve the activation of ERK1/2 and PI3K-AKT, leading to cell survival and cell growth pathways. Created with BioRender.com.

The main mechanosensors of cell–ECM components interactions are integrins. Cardiac myocytes mostly express α1β1, α5β1, and α7β1, which mainly bind collagen, fibronectin, and laminin, respectively. Integrin conformational changes cause the activation of downstream integrin-mediated signaling cascades and the recruitment of multiprotein complexes to focal adhesions [73,79] (Figure 1).

Since integrins lack enzymatic activity, the activation of downstream signaling factors requires interactions with kinase proteins. When ECM ligands (collagen, laminins, fibronectin) bind to integrins, adaptor proteins such as vinculin, paxillin, and talin, as well as FAK, ILK, and Src, are recruited [80,81]. Interestingly, integrin α subunits generally facilitate adhesion and give ECM ligand specificity, which consequently induces conformational changes and integrin clustering, finally recruiting downstream molecules, whereas the integrin subunit β1, which directly binds to cytoplasmic adaptors and signaling molecules, has been shown to have a key role in coupling mechanical stretch to the activation of downstream effectors (e.g., MAPKs, Rho GTPases, FAK/Src) [80,81,82]. As a result of integrin clustering and integrin-mediated ECM mechanosensing, intracellular events involving FA complex formation, actin polymerization, and, finally, actin–myosin stress fiber formation, provide the mechanosensitive link between the extracellular and intracellular environments, as well as rigidity to the cell [82].

Intriguingly, integrins can modulate ion channels, including L-type Ca^2+^ channels (LTCC) [80,83,84,85]. In a recent study, Okada and colleagues demonstrated that the overexpression of integrin α7β1 protected cardiac myocytes from ischemia/reperfusion (I/R) injury by modulating intracellular mitochondrial Ca^2+^ overload. Moreover, they showed that the integrin β1 subunit can interact with and stabilize ryanodine receptors 2 (RyR2) in an ECM-dependent manner [80,86].

Other players in mechanoelectric feedback are the mechanosensitive ion channels (MSCs) or stretch-activated channels (SACs), which are involved in the regulation of ion voltage in a mechanoelectric loop in response to mechanical stress [87]. One important family of SACs is the transient receptor potential vanilloid type 2 (TRPV2) channels, which are involved in ion regulation in response to mechanical stress. Interestingly, Iwata and colleagues demonstrated that TRPV2 was overexpressed and hyper-activated in dilated cardiomyopathy (DCM), leading to excessive Ca^2+^ influx [88,89].

Furthermore, it is well known that angiotensin II type I receptor (AT1R), a transmembrane-spanning G protein-coupled receptor (GPCR), is involved in mediating mechanical stimuli by activating different pathways. AT1R activation by angiotensinogen II activates the canonical Gαq protein signaling pathway, which leads to inositol trisphosphate (IP3) and diacylglycerol (DAG) synthesis, the regulation of intracellular Ca^2+^, and downstream kinases (i.e., ERK1/2) activation [82,90]. Stretch-induced AT1R activation triggers conformational changes in β-arrestin, selectively stimulating receptor signaling in the absence of G protein activation. In addition to activating direct effectors such as G protein and β-arrestin, AT1R mechanosensing promotes downstream pathways involved in altering the ECM, gap junction formation, and ion channel functionality [90,91,92,93].

Another important mechanosensory complex is dystroglycan, which links laminins in the ECM to the actin cytoskeleton through dystrophin. Mutations in the dystrophin–dystroglycan complex lead to the impaired mechanical activation of nitric oxide signaling in cardiac muscle [79,94].

Alterations in extracellular membrane stiffness can lead to different adaptive responses, the most dramatic of which is the disassembly of FAs [15]. A study by Shi and colleagues evaluated the two fundamental proteins of the adhesion complex, paxillin and vinculin, through the use of a dynamic cell culture system based on polymers capable of reproducing a surface ranging from flat to rough [15]. This study revealed that these proteins are capable of reacting to rapid changes by modifying their affinity to the extracellular matrix through the initial disassembly of FAs and subsequent regeneration of the adhesion, which is restored once the tissue returns to the initial relaxed state [15]. Interestingly, the structural module proteins (talin, vinculin, and tensin1), but not the signaling module proteins (FAK and paxillin), modify their turnover in response to ECM stiffness [52,53]. This suggests that FA proteins are involved in linking integrins to the actin cytoskeleton and are directly involved in sensing ECM mechanical characteristics [52]. Therefore, to properly work, the process of mechanotransduction requires the cooperation of both modules: (i) the structural proteins are involved in directly sensing mechanical stimuli (mechanosensing), whereas (ii) the signaling module proteins are involved in generating the intracellular signaling events in response to these forces (mechanosignaling) [95].

In cardiomyocytes, in particular, the importance of a solid structure must also be accompanied by flexibility to allow for efficient contraction [96]. One fundamental element is the protein nebulette. In fact, nebulette allows FAs to withstand mechanical stress by interacting with intermediate desmin filaments, maintaining elasticity to enable cardiomyocytes to function even under dynamic conditions [97]. The ability of the ECM to withstand mechanical stress is also enabled by the presence of laminin and fibronectin [98], which, in addition to promoting cell adhesion, also induce the expression of normal levels of FAK. A decrease in FAK levels would result in reduced resistance to mechanical stimulation [98].

Vinculin, which is one of the major sensors activated through FAs, works closely with the muscular variant, metavinculin [99]. It has been shown that these two matrix proteins are essential for maintaining the normal adhesion structure; indeed, any ablation of these proteins predisposes to an increased susceptibility to changes caused by external forces [100]. Under normal conditions, cardiomyocytes cope with mechanical stresses by remodeling matrix anchorages, but this mechanism is ineffective if alterations in the described proteins are present. Specifically, two particular cases have been analyzed: one with heterozygous vinculin ablation (VIN +/−), in which normal cardiac development has been demonstrated—unless stress events such as hypertension, obesity, and diabetes occur—and another case with homozygous ablation (VIN −/−), which is associated with a lethal condition [99]. Consequently, it can be stated that if a person with heterozygous vinculin defects undergoes stress, there is a change in focal contacts and the onset of heart diseases [99], thus demonstrating the importance of this protein in the mechanism of response to the mechanical characteristics of the matrix. Vinculin, however, plays another important role in the mechanosensing mechanism: it is capable of perceiving intracellular tension generated by mechanical stress such as tension or torsion and regulating intracellular signals accordingly [52,101,102]. Previous work by Carton and colleagues demonstrated that during the differentiation of H9c2 cardiomyocytes, there is an increase in vinculin expression and its recruitment to the cell membrane in differentiated cells, leading to the strengthening of integrin-based ECM adhesion complexes. In addition, the distribution of vinculin along FAs undergoes alterations after applying mechanical stress, leading to changes in FA length. These findings highlight a correlation between FA formation, cardiomyocyte differentiation, and mechanotransduction [103].

Moreover, a study by Yamashita and colleagues [104] revealed that vinculin detects changes in ECM stiffness. In this study, cells cultured on stiffer substrates exhibited an increased number and length of FAs compared to those on softer substrates. They also showed that mutations in the binding site of vinexin-α, an FA protein that interacts with vinculin through its SH3 domains [104,105,106], and that the depletion of vinculin or vinexin-α result in a prevented stiffness-dependent increase in cell velocity observed in wild-type cells [105]. These results highlight how vinculin, associated with vinexin -α, senses ECM stiffness and subsequently transmits signals through paxillin and FAK to regulate cell motility.

Chorev and colleagues [107] demonstrated that vinculin interacts with Arp2/3 but not with the whole Arp complex, regulating FA maturation through a “hybrid complex”. This complex, formed by the nucleation module and the anchoring module, is responsible for actin polymerization and branching polymerization [108]. Interestingly, in the presence of a specific mutant vinculin that is unable to bind to other ligands such as the Arp2/3 complex, the vinculin mutant disrupts actin binding and reduces the ability of cells to spread, adhere, and sustain traction forces [101,108]. All these molecular mechanisms of mechanotransduction have been underlined and confirmed by several in vivo studies [39,42,109,110,111,112,113,114].

A study performed on zebrafish models demonstrated that the mechanical forces generated by cardiac contractility were able to regulate the F-actin rearrangement, thus allowing for cardiomyocyte myofilament maturation through the vinculin VCL–SSH1-CFL axis. These researchers also found that vinculin localization and activation are regulated by cardiac contractility and blood flow and that vinculin is essential for cardiomyocyte myofilament maturation [114]. Consistently with these studies, the knock-down of vinculin in human mesenchymal stem cells (MSCs), differentiated towards a muscle lineage, resulted in a reduction in the expression of myoD and in the subsequent differentiation of the cells to muscle lineage [115].

Notably, mutations in the vinculin isoform gene have been identified in cardiac hypertrophy (HCM) and dilated cardiomyopathies (DCMs) [116,117,118]. In HCM, which is characterized by abnormal thickening of the left ventricle and associated contractile impairments, vinculin mutations enhance mechanical alterations within the myocardium [116,118,119]. Conversely, in DCM, which is characterized by abnormal dilation of the heart muscle and subsequent contractile dysfunction, vinculin mutations play a role in the pathogenesis, together with other genetic anomalies involving nebulette, integrin-linked kinase (ILK), or talin [116,117,118,120].

Furthermore, alterations in the gene encoding for titin, another FA protein, have been reported in HCM, DCM, and restrictive cardiomyopathy (RCM), which is characterized by ventricular wall stiffness [118,121,122,123]. Alongside gene modifications, alterations in integrin expression have also been observed in cardiomyopathies and ventricular cardiac hypertrophy, where structural remodeling of the heart muscle occurs in response to imbalanced mechanical stresses [124,125].

Collectively, these findings underscore the crucial significance of FAs and their involvement in mechanosensing and mechanosignaling pathways during pathophysiological processes. However, a comprehensive understanding of how these mutations modulate mechanosensing and mechanosignaling pathways remains to be deeply investigated. Further investigations are essential to unravel the complex interplay between genetic aberrations, mechanical cues, and signaling cascades, with the ultimate goal of understanding the complexities of cardiac pathologies and identifying novel therapeutic targets.

## 4. Focal Adhesion-Mediated Mechanosignaling in Cardiac Muscle

From a signaling pathway perspective, cardiomyocytes respond to matrix stiffness by modifying the expression of cardiac development agonists such as components of the PI3K/AKT or p38/JNK pathways; furthermore, they alter the interactions among various proteins involved in the adhesion complex, which, in response to these modifications, adjust their affinity to the matrix [14]. As a matter of fact, structural proteins modify their activated state and turnover rate, allowing for the signaling of substrate stiffness changes [95]. During cardiomyogenesis, the Wnt/β-catenin signaling cascade drives cytoskeletal organization and contractility, and this pathway may be modulated by mechanical forces. In addition, integrins and integrin-associated proteins sense mechanical forces (both active and passive) generated during cardiac development and enhance tissue stiffening, alter gene expression, activate soluble downstream pathways, and assist in structural reorganization. Intriguingly, integrin expression is upregulated in response to mechanical stimuli, improving cell adhesion and FA assembly in order to modify and extend cardiomyogenesis, indicating an interesting role of FAs during cardiac differentiation [18,103,126,127].

**Figure 2:**
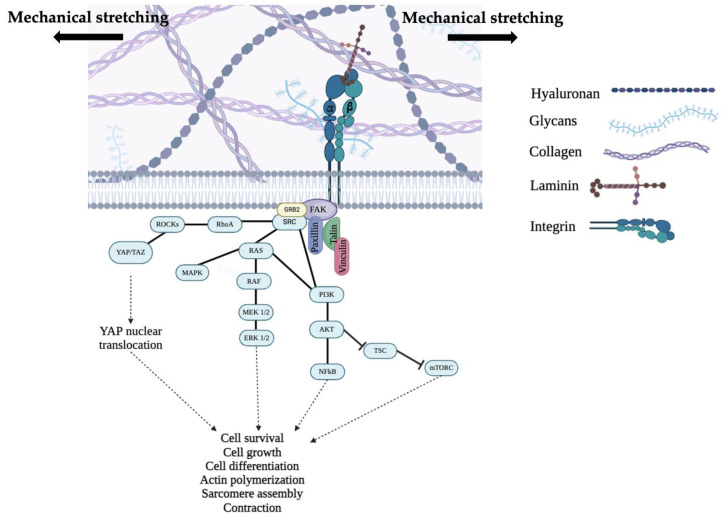
Schematic representation of mechanical stress-mediated pathways, leading to cell survival, cell growth, cytoskeletal organization, and cardiac myocyte contraction. Created with BioRender.com.

Studies in the literature suggest that the FAK/Src complex mediates physiological cardiac hypertrophic growth and survival signaling after a mechanical load, leading to the activation of some pathways, such as the Ras cascade, NF-kβ activity, MAPK/ERK signaling, and hippo pathways (Figure 2) [78,128,129,130]. It is known that FAK autophosphorylation recruits Src, which, in turn, enhances FAK phosphorylation and, thus, FAK activity, activating different downstream pathways [131,132]. Interestingly, Torsoni and colleagues [133] demonstrated that stretch-induced FAK translocation and clustering from the perinuclear area to myofilaments is dependent on the autophosphorylation of Tyr-397, which recruits and activates Src family kinases [131,133,134]. Moreover, they demonstrated that by disrupting the stretch-induced activation of FAK/Src signaling, the stretch-induced Fak aggregation at NRVM myofilaments was withdrawn [133]. Hence, the inhibition of Tyr-397 autophosphorylation would prevent Fak/Src clustering at costameres within cardiac myocytes [131,133,134].

The YAP/TAZ complex, related to non-canonical hippo pathways, has an interesting role in cellular mechanics. YAP/TAZ are the terminal effectors of the hippo signaling pathway and act as transcriptional cofactors with other DNA-binding proteins to regulate cell survival, cell proliferation, and, finally, organ development and growth. The YAP/TAZ complex is regulated by actin, in this case, actin filaments and, more specifically, their conformation and tension, which influence the nuclear translocation. The hippo pathway is also involved in heart development, cardiomyocyte apoptosis after myocardial infarction, and hypertrophic and dilated cardiomyopathies [18,135]. Moreover, the dissociation of FAK from Shp2 in response to substrate rigidity activates the AKT/TSC2/mTOR and ERK1/2 pathways, which promote the protection of cardiomyocytes from apoptosis, mediating the activation of the anti-apoptotic element NF-kβ [98,136]. Specifically, NF-kβ in cardiomyocytes is recruited following the activation of Tumor Necrosis Factor-alpha (TNFα), interleukin 1 (IL-1), and interleukin 6 (IL-6), as well as antagonists of G protein-coupled receptors such as angiotensin 2, phenylephrine, or endothelin. The presence of stress events leads to an increase in NF-kβ in the nucleus and its DNA binding activity, allowing for the processing of a stress response that ensures cardiomyocyte survival by maintaining the correct structure of focal contacts [136].

It is known that the phosphorylation of FAK has multiple crucial roles: in response to stress conditions, FAK accumulates in the nucleus, where it interacts with the transcription factor MEF2 through the MADS-box domain and Focal Adhesion Target (FAT) domain of FAK [137,138]. This interaction positively regulates c-jun expression in cardiomyocytes, leading to the adaptation of FAs during a sustained mechanical stretch [138]. The presence of external forces, therefore, activates various cardiac responses, including the activation of Mitogen-Activated Protein Kinases (MAPKs) and ROCKs by FAK [139]. FAK kinase activation leads to the recruitment of the adapter Grb2, which activates the MAPK cascade through interaction with a member of the Ras family, resulting in an adaptive response by cardiomyocytes, which, in response to external forces, tend to increase their elasticity at adhesion points [140].

In conclusion, given the different mechanisms by which cardiomyocytes respond to substrate rigidity or mechanical stress characteristics, it is clear how the remodeling of a distinctive structural element is essential to maintain the mechanical properties of the cardiac phenotype (Table 2). As demonstrated, focal contacts, undergoing modifications in their protein structure through the activation of different pathways and thanks to their adaptability to various events, allow cardiomyocytes to maintain their role as contractile cells of the myocardial tissue even under defined unfavorable conditions.

## 5. Conclusions

Due to myocardial contraction, cardiomyocytes are subjected to constant mechanical deformation. The maintenance of physiological cardiac stiffness emerges as a critical environmental cue, influencing both overall ventricular diastolic function and myocardial mechanical properties. This review emphasizes the complex regulation of FAs in cardiac commitment and mechanotransduction. In fact, inside-out and outside-in signaling pathways, activated in response to changes in matrix stiffness, contribute to downstream mechanosensitive signaling cascades in cardiomyocytes. While the general mechanisms of mechanotransduction have been identified, understanding their implications in pathological contexts and their potential translation into therapeutic targets remains an ongoing challenge. Hence, a deeper understanding of the roles played by intra- and extracellular molecular modifiers in influencing tissue compliance is crucial, such as gaining knowledge on how various cardiac cell types modulate ECM stiffness, especially in the context of collagen deposition within diseased or injured cardiac tissue. These studies will enhance the understanding of how alterations in ECM stiffness and FA-related pathways impact the affected myocardium during the initiation and progression of cardiac disease, as well as functional repair after damage. Such insights hold the potential to improve cardiomyocyte performance and induce regenerative processes without compromising the physiological stiffness of the ventricular wall. The primary aim will be to unravel novel therapeutic strategies for cardiac injuries, addressing the complex interplay between mechanical cues and myocardial health and regeneration.

## Figures and Tables

**Table 1:** Key concepts and main interplayers in cardiac differentiation.

Highlights	Proteins Involved	Process Sustained	References
Cardiomyocyte Myofibrillar Assembly	NMM II, α-actinin-2 fibers Titin, α- and/or β-cardiac myosins	Pre-myofibril composition. Final myofibril composition.	[22]
Sarcomere Remodeling during Heart Development	Myosin, actin, titin (sarcomeric proteins)	Influence the contractile properties of cardiomyocytes and force generation.	[28,30]
	Titin	Provide elasticity and contribute to the passive stiffness of cardiomyocytes.	[31,32]
	β-cardiac myosin	Direct sarcomere assembly by generating the required basal tension.	[31]
Focal Adhesion Signaling and Cardiomyogenesis	Integrin/FAK/PI3K-P85 pathway	FA’s maturation process in cardiac differentiation.	[35,36,37,38]
	FAK-Tyr397 phosphorylation	Myocardial development, activate survival-promoting pathways.	[45,46,47]
Proteins Involved in FA Maturation	Mint1/Veli/SAP97/CASK complex	Structural and functional support to the sarcomere.	[48]
	VEGF	Enhance the adhesion of cardiomyocytes to the ECM.	[49]

**Table 2:** This summary table captures the key points discussed in the text, providing an overview of the mechanisms and responses related to cellular mechanics and mechanotransduction in cardiomyocytes.

Highlights	Main Players	Process	References
Forces Affecting Cardiomyocytes	Contractions, hemodynamic pressure, ECM-related passive elasticity	Changes in laminin, collagen, matrix protease, and proteoglycan expression.	[68,69]
Focal Adhesion proteins involved in mechanotransduction	FAK/Src complex	Cardiac hypertrophic growth and survival signaling.	[15,78]
	FAK	Adaptive responses via MAPK and AKT/TSC2/mTOR pathways.	[78,137]
	Vinculin	Regulation in FA maturation.	[101,107]
	Talin, vinculin, tensin1	Structural module.	[52,53]
	FAK, paxillin	Signaling module.	[52,53]
Signaling Pathways in Response to Matrix Rigidity	PI3K/AKT, p38/JNK pathways	Regulation of interactions between adhesion complex and structural proteins.	[14]
	Wnt/beta-catenin signaling	Cytoskeletal organization, regulation of contractility during cardiomyogenesis.	[14,95]
	Non-canonical Hippo pathway through YAP/TAZ	Heart development, cellular mechanics.	[18,135]
	AKT/TSC2/mTOR and ERK1/2 pathways	Prevent cardiomyocytes’ apoptosis.	[98,136]
	NF-kβ	Cell survival, correct assembling of FAs.	[136]

## Data Availability

Not applicable.
